# Data showing the effects of geotechnical properties of lateritic soil mixed with coconut shell powder in Ado-Ekiti, south western Nigeria

**DOI:** 10.1016/j.dib.2019.103861

**Published:** 2019-04-05

**Authors:** Ifetayo J. Oluwafemi, Timothy O. Laseinde, Joshua Akinwamide

**Affiliations:** a Postgraduate School of Engineering Management, University of Johannesburg, Auckland Park Kingsway Campus, Johannesburg, 2006, South Africa; b Department of Mechanical Engineering Science, University of Johannesburg, Auckland Park Kingsway Campus, Johannesburg, 2006, South Africa; c Department of Geotechnics, Federal Polytechnic Ado Ekiti, Ekiti State, Nigeria

**Keywords:** Coconut shell powder, Cement, Substituents, Soil

## Abstract

In this data, the effect of locally available additive Coconut shell powder (CSP) substitute in determining the geotechnical properties of Ado Ekiti soil was assessed. The samples were collected from two borrow pits, Ado-Ijan road and Ado-Ilawe road, at two points each, and were treated using substitute of coconut shell powder, considering several percentages of its content at 0%, 2%, 4%, 6%, 8% and 10%. Laboratory tests such as specific gravity, consistency limit test, grain size distribution test, compaction test, CBR test, triaxial compression test, permeability test was conducted on the soil samples collected, and untreated soil was determined. The summary shows that the liquid limit, plastic limit, Plasticity Index, Maximum Dry Density and Optimum Moisture Content increases with increase in percentage of the additive and later decrease at higher percentage of additive, mostly at 8% and 10% respectively. The value of California Bearing ratio in percentage increases as the percentage of additive increases, although at 0% additive, the soil does not meet specification of road construction and the value of permeability ‘k’ also increases. The summary of triaxial test also shows increase in the strength properties of the soil at certain percentage likewise, the summary of unconfined compressive strength shows same. This indicates that the additives improves the geotechnical properties of the soil samples to a certain percentage and conversely, have a negative effect on it at a higher percentage 8% and 10% respectively.

**Table:** Specifications table

Subject area	Civil Engineering
More specific subject area	Construction material and Waste Management
Type of data	Figures, graphs and table
How data was acquired	Coconut shell was extracted from coconut. The extracted dried coconut shell was grinded into powder using disc milling machine.
Data format	Raw and Analysed
Experimental factors	Four soil samples taken from two borrow pits, i.e. two from Ado-Ijan Road (pit A & pit B) and the other two from Ado-Ilawe Road (pit A & pit B), Ado-Ekiti were disturbed samples. The field assessment was based on investigation which involved mapping the road alignment and observing the rock and soil conditions and the general nature of the environment where the road is routed. Coconut shells obtained from the market at Ado-Ekiti mixed with the processed soil samples were subjected to the following laboratory tests: Specific gravity, Consistency limit tests, Grain size distribution tests, Compaction test, CBR test, Triaxial compression tests, Unconfined compressive strength test, Permeability test
Experimental features	Coconut shell powder was adopted as an additive in stabilizing the soil to predict the Engineering properties of the soil and its performance under field conditions
Data source location	Federal Polytechnic Ado Ekiti, Ekiti State, Nigeria.
Data accessibility Related research article	The data is within this article Ikumapayi OM, Akinlabi ET. Data showing the effects of vibratory disc milling time on the microstructural characteristics of Coconut Shell Nanoparticles (CS-NPs). Data in brief. 2019 Feb 1; 22:537–45 [1].

**Table:** 

**Value of the data** • Data can be used to examine the properties and performance of coconut shell powder as alternative material for soil stabilization. • Data can be useful for researchers in comparing strength properties of coconut shell powder with other additive useful waste materials (rice hush ash) [2], [3], [4] strength properties for soil stabilization. • The test data shows that Coconut shell powder can be used as a biological material for metal removal and recovery technologies. • The test data allows for investigation on the use of coconut shell as another possible material for stabilization material for the reinforcement in concrete. • The data allows for further investigation on the use of Coconut shell powder as additive for stabilizing of Lateritic soil in road construction. • The test data presented can be useful for construction engineers in appropriating the accurate percentage of Coconut shell powder as an addictive for soil stabilization.

## 1 Data

**Fig. 1:**
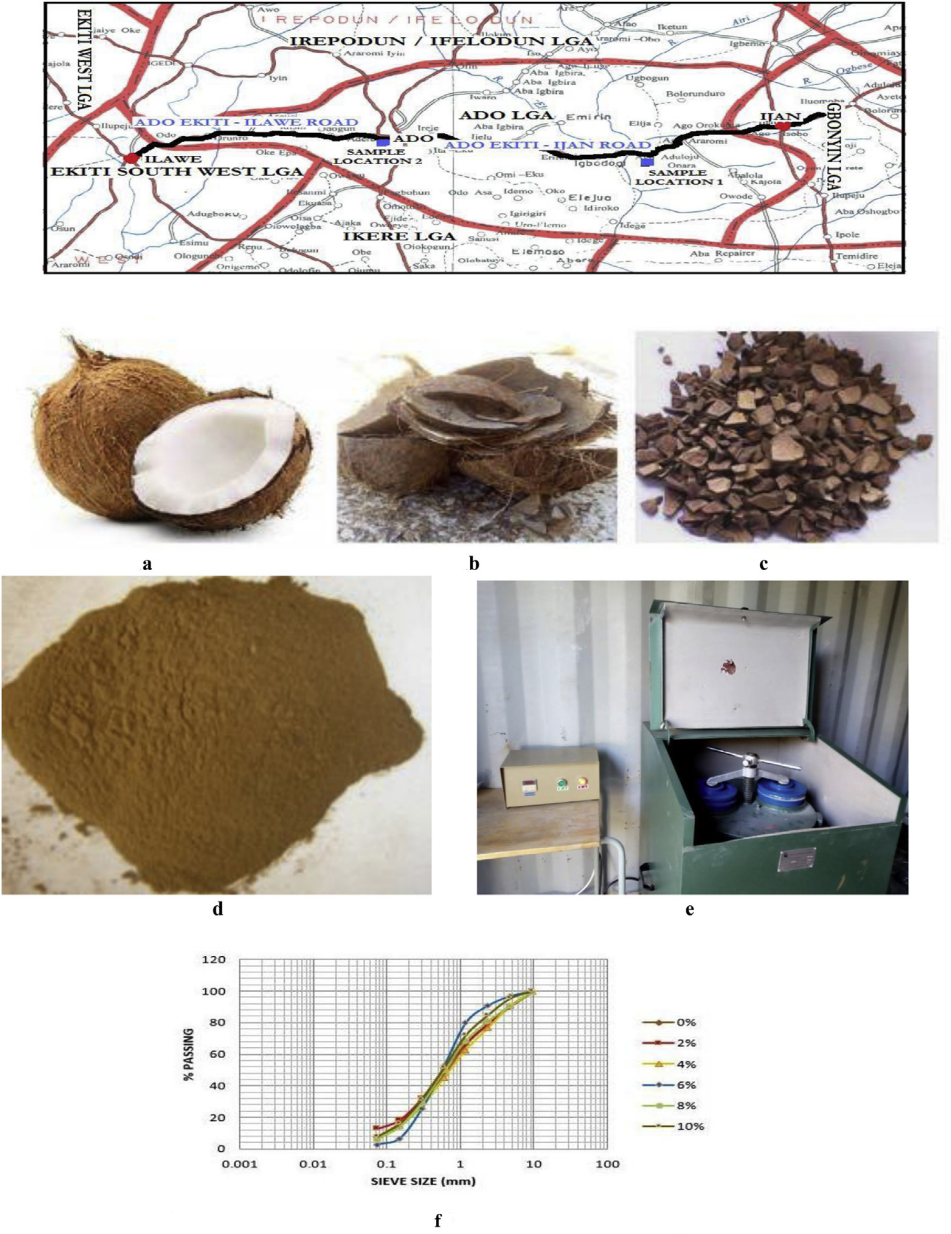
Location of the sampling area. **a.** Coconut fruit. **b**. Coconut shell. **c**. Broken Coconut shell. **d.** Coconut shell Powder (CSP). **e.** Disc Milling Machine. **f**. Grain size distribution curve for Ado-Ijan pit A.

The data presented was obtained from four soil samples taken from two borrow pits, i.e. two from Ado-Ijan Road (pit A & pit B) and the other two from Ado-Ilawe Road (pit A & pit B), Ado-Ekiti, Ekiti State, Southwestern part of Nigeria (Fig 1). and analysed in the geotechnics laboratory of Federal Polytechnic Ado Ekiti, Ekiti State, Nigeria. Fig. 1a–d shows the procedure of obtaining the coconut shell powder using digital disc milling machine Fig 1e.

## 2 Experimental design, materials, and methods

Soil samples portions were taken using basic tools namely shovel, digger, and Polythene bags for soil storage. Soil samples collected were taken to the laboratory where sample portions were tested to determine the moisture contents, and the rest were sun dry for two weeks. The coconut shells collected were sun dried for over two weeks to achieve sufficient dehydration. They were broken into pieces by pounding in mortar, and were later taken to a pulverization machine, to break them into smaller pieces. After it has been pulverized, it was milled and turn into powdery form using a disk milling machine. Coconut shell powder was added to each of the soil samples in 2, 4, 6, 8 and 10% by weight of the samples before subjecting it to the following preliminary tests: Specific gravity, Consistency limit tests, Grain size distribution tests, Compaction test, CBR test, Triaxial compression tests, Unconfined compressive strength test, and Permeability test. All tests and geological properties of the soil were determined in accordance with B.S.1337 [5].

### 2.1 Specific gravity data

Table 1 shows that the specific gravity for both Ado-Ijan pit A and pit B varies from 2.56 to 2.65, and that of Ado-Ilawe. Pit A and pit B varies from 2.01 to 2.05 respectively. With the addition of coconut shell powder ‘CSP’ as the additive, the samples from Ado-Ilawe varies from 2% to 10%, and Ado-Ijan road samples varies from 2.09 to 2.51. This implies that the addition of the additive shows various variation in the specific gravity of the soil. The Ado-Ilawe sample which varies from 1.65 to 2.48 with the addition of the additive, shows the different variation in the specific gravity of the soil samples.

**Table 1:** Summary of the specific gravity test.

Location		Additive (%)	Specific gravity (SG)
Ado-Ijan	Pit A	0	2.56
		2	2.21
		4	2.30
		6	2.44
		8	2.39
		10	2.33
	Pit B	0	2.65
		2	2.09
		4	2.39
		6	2.44
		8	2.51
		10	2.32
Ado-Ilawe	Pit A	0	2.05
		2	1.76
		4	2.38
		6	2.35
		8	2.41
		10	2.25
	Pit B	0	2.01
		2	1.65
		4	2.30
		6	2.48
		8	2.31
		10	1.99

### 2.2 Grain size distribution

**Fig. 2:**
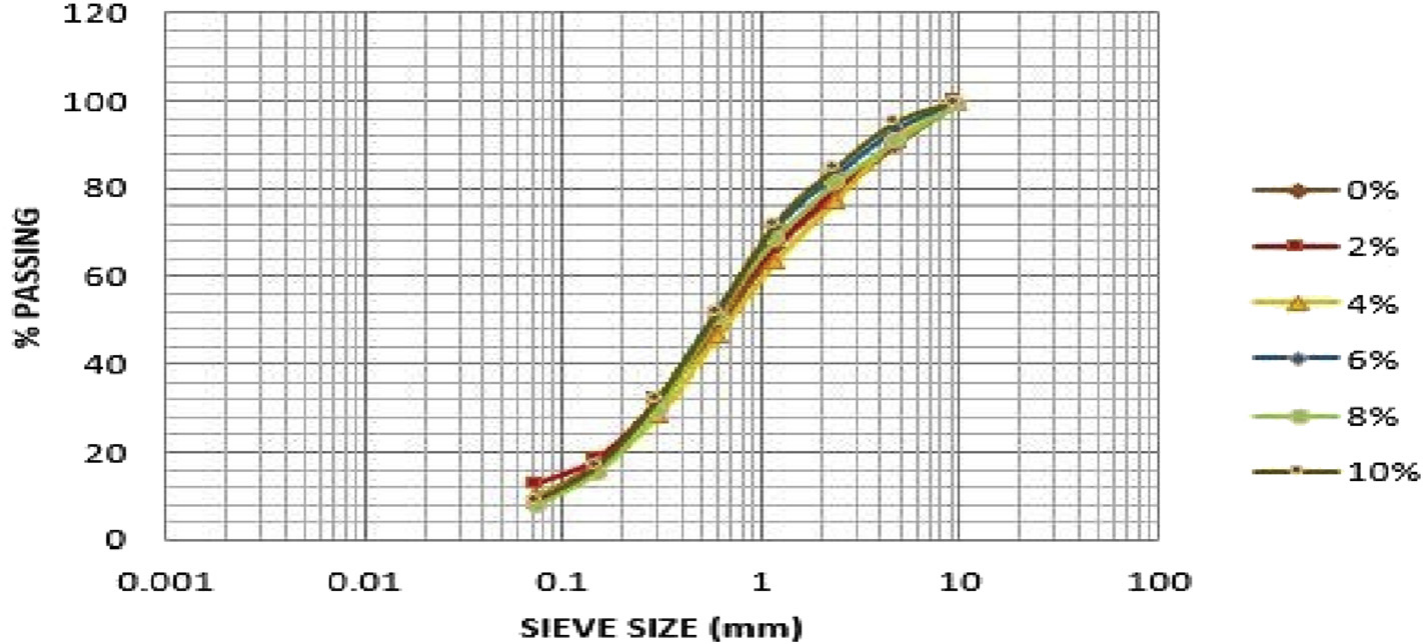
Grain size distribution curve for Ado-Ijan pit B.

**Fig. 3:**
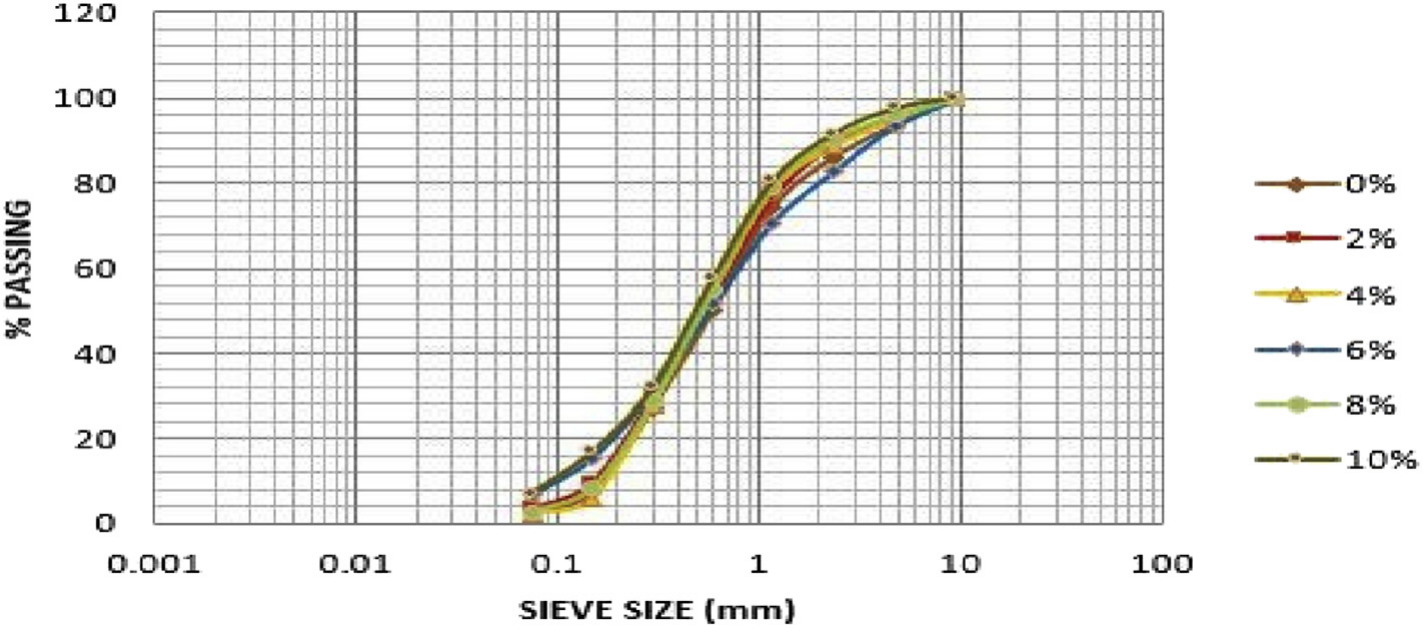
Grain size distribution curve for Ado-Ilawe pit A.

**Fig. 4:**
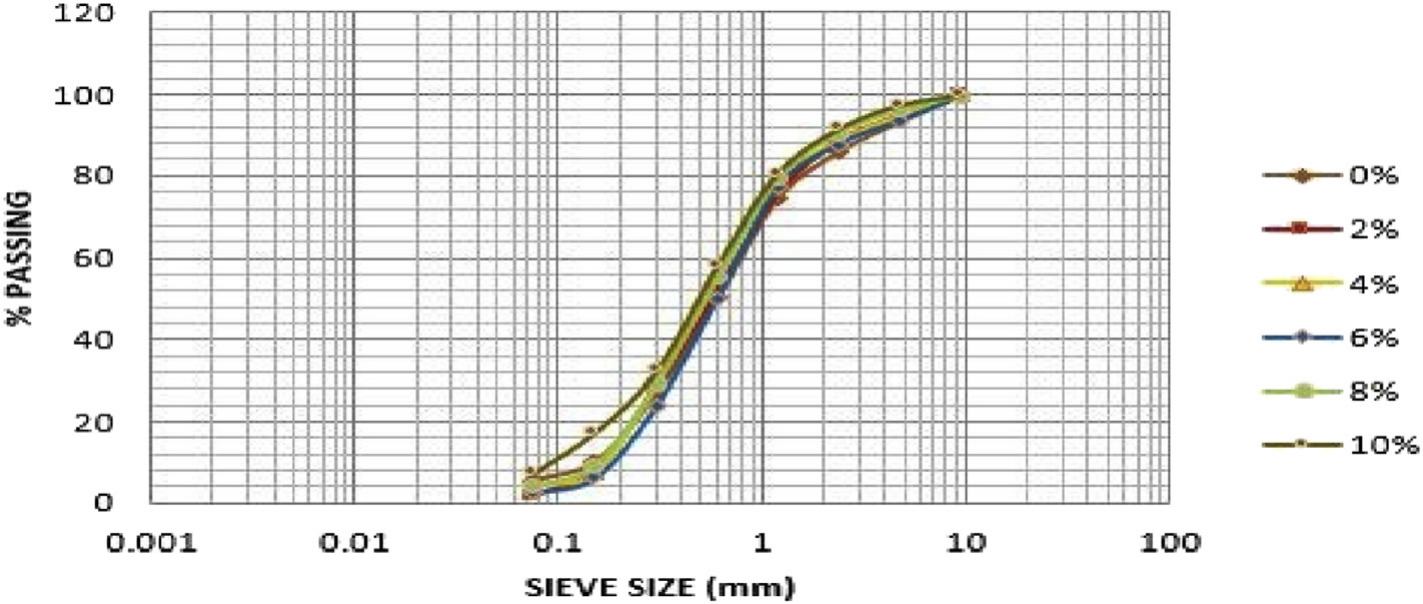
Grain size distribution curve for Ado-Ilawe pit B.

Table 2 shows that the soil sample from Ado-Ijan pit A at 0% of additive has higher percentage of sand i.e. 71.49 to 20.93% of gravel and 7.58% of fine, which can be classified as clayey soil in accordance with unified soil classification system USCS (i.e. C =Clayey;>12% fines, PI > 7). While with the addition of additives, the percentage of sand varies from 66.41% to 76.98%, the same trend was observed in pit B. For Ado-Ilawe pit A at 0% it shows that the percentage of sand is also higher than gravel and fines having 83.13% sand. According to (ASTM D422-63 (2007) Standard Test Method for Particle-Size Analysis of Soils) determined by mechanical method, percentage retained on sieve 2.36mm is classified as percentage of gravel, percentage passing sieve 75 mic is classified as a percentage of fines (clay or silt) and summation of both subtracted from 100% is termed as percentage of sand. Fig. 1, Fig. 2, Fig. 3, Fig. 4 shows the plots of the graph below. Table 2 shows the summary of grain size distribution tests.

**Table 2:** Summary of the grain size distribution tests.

Location		Additive content (%)	Percentage of gravel	Percentage of sand	Percentage of fines (Silt-Clay)
Ado-Ijan	Pit A	0	20.93	71.49	7.58
		2	20.69	66.41	12.90
		4	23.42	69.00	7.58
		6	17.29	76.45	6.62
		8	19.04	75.06	5.90
		10	15.06	76.98	7.96
	Pit B	0	21.37	69.14	9.49
		2	19.96	67.24	12.80
		4	22.85	67.50	9.65
		6	16.95	74.25	8.80
		8	18.79	73.34	7.87
		10	15.49	75.64	8.87
Ado-Ilawe	Pit A	0	14.06	83.13	2.81
		2	10.98	85.63	3.31
		4	11.51	86.29	2.20
		6	9.37	87.87	2.76
		8	9.84	87.45	2.71
		10	8.46	84.55	6.99
	Pit B	0	14.00	80.43	5.57
		2	11.26	86.99	1.75
		4	11.29	84.50	4.21
		6	12.23	85.22	2.55
		8	9.50	86.46	4.04
		10	8.36	84.15	7.49

### 2.3 Consistency limits

**Fig. 5:**
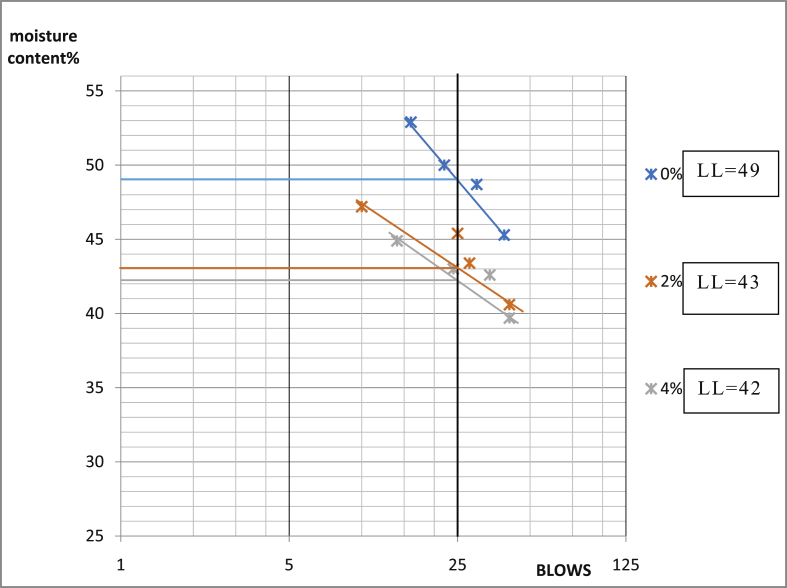
Consistency limits graph Ado-Ilawe pit A.

**Fig. 6:**
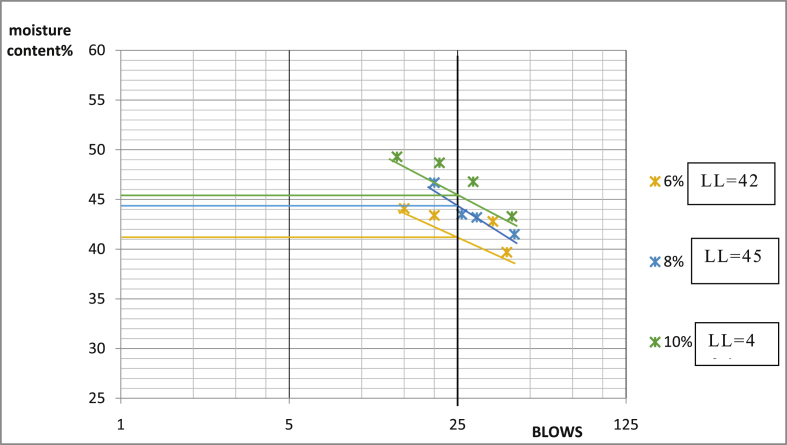
Consistency limits graph Ado-Ilawe pit A.

**Fig. 7:**
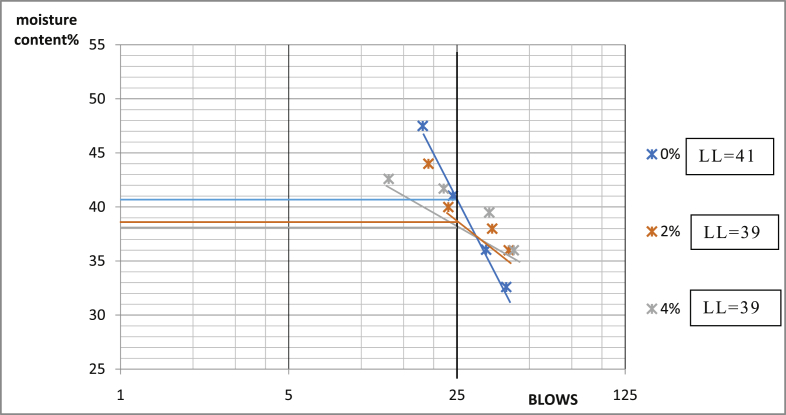
Consistency limits graph Ado-Ilawe pit B.

**Fig. 8:**
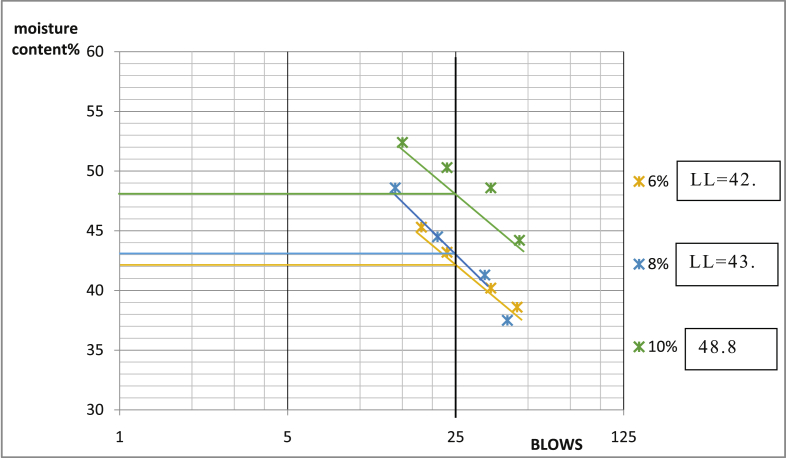
Consistency limits graph Ado-Ilawe pit B.

**Fig. 9:**
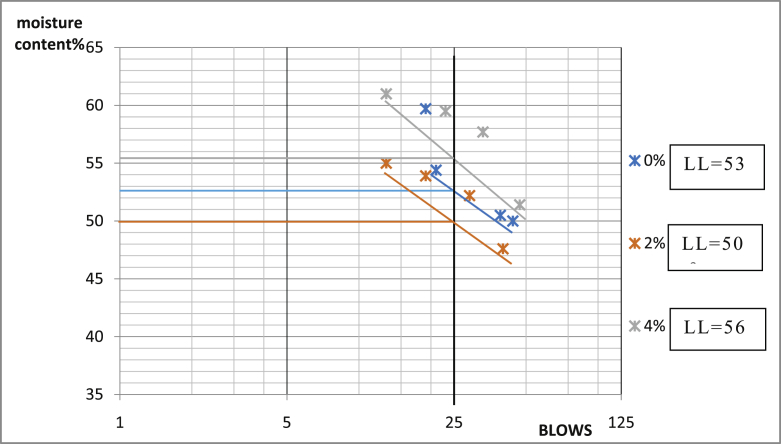
Consistency limits graph Ado-Ijan pit A.

**Fig. 10:**
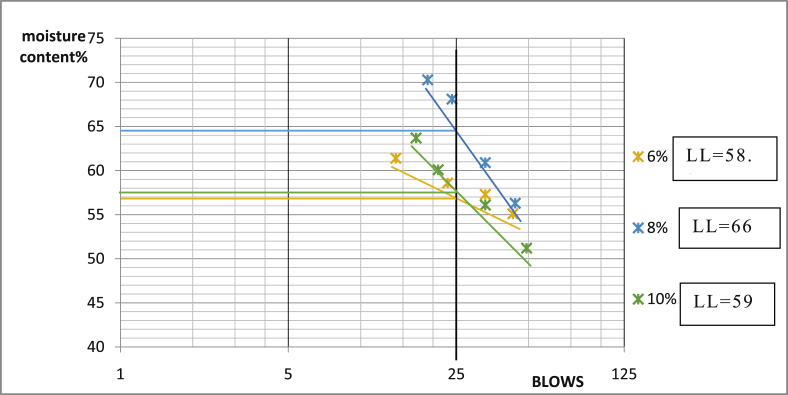
Consistency limits graph Ado-Ijan pit A.

**Fig. 11:**
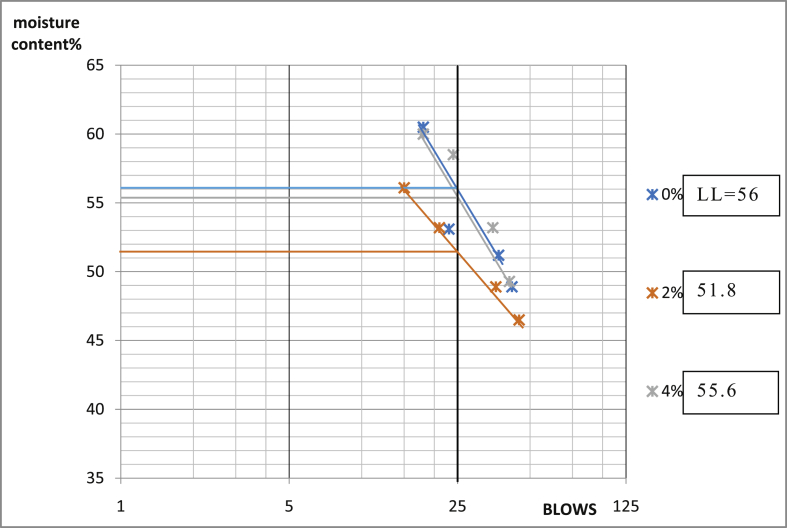
Consistency limits graph Ado-Ijan pit B.

**Fig. 12:**
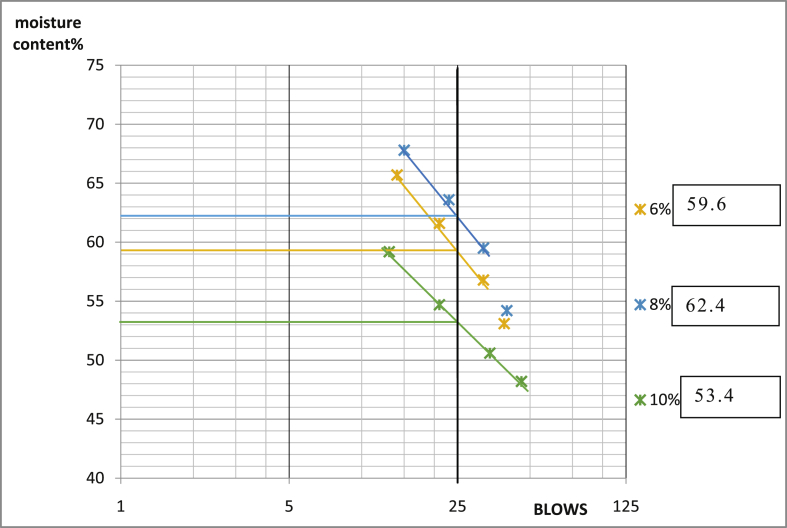
Consistency limits graph Ado-Ijan pit A.

Table 3 shows that Ado-Ijan Road with the addition of the additive (CSP) increases the Plasticity Index PI from 22.3 to 38.8, whereas the Plasticity Index for 0% additive is 24.0, the same trend is observed in Pit B. Also, for Ado-Ilawe Pit A show that the Plasticity Index for 0% additive is 22.5 and when mixed with additives it varies from 12.5 to 22.5; furthermore, it is also observed that increment in percentage of additive increases the linear shrinkage of the soil (Shrinkage Limit). Fig. 5, Fig. 6, Fig. 7, Fig. 8, Fig. 9, Fig. 10, Fig. 11, Fig. 12 shows the graph of consistency limits.

**Table 3:** Consistency limits.

Location		Additive content (%)	Liquid limit (LL)	Plastic limit (PL)	Plasticity index (PI)	Shrinkage limit (%)
Ado-Ijan	Pit A	0	53.5	29.5	24.0	6.0
		2	50.9	28.6	22.3	6.0
		4	56.2	28.3	27.9	6.4
		6	58.3	26.1	32.2	7.1
		8	66.0	27.2	38.8	8.2
		10	59.0	27.7	31.3	9.0
	Pit B	0	56.4	30.6	25.8	5.9
		2	51.2	28.6	23.2	6.2
		4	55.6	27.9	32.7	6.5
		6	59.6	26.9	32.7	7.0
		8	62.4	27.2	35.2	7.6
		10	53.4	26.9	26.5	8.7
Ado-Ilawe	Pit A	0	49.0	26.5	22.5	4.0
		2	43.0	27.9	20.1	4.3
		4	42.2	29.7	12.5	5.0
		6	42.0	23.2	18.8	5.4
		8	45.0	22.5	22.5	6.0
		10	46.1	28.3	17.8	6.8
	Pit B	0	41.5	27.5	14.0	4.0
		2	39.4	25.5	13.9	4.2
		4	39.0	25.0	14.0	4.7
		6	42.8	22.8	20.0	5.5
		8	43.8	22.9	20.9	6.1
		10	48.8	28.3	20.5	6.7

### 2.4 Compaction test

Table 4 shows the details of compaction tests data with maximum dry density (MDD) as well as conforming optimum moisture content OMC of sun-dried soil compacted using west Africa method. In pit A for Ado-Ijan road, MDD increases from 2005Kg/m3 to 2191Kg/m3 with the addition of the additive coconut shell powder CSP, while it is 1786Kg/m3 for an ordinary soil sample. The same trend is also observed in pit B of the same location. Also, in pit A for Ado-Ilawe the MDD increases 1993(Kg/m3) to 2164(Kg/m3) with the addition of CSP, the same trend is also observed in pit B of the location.

**Table 4:** Summary of the compaction test.

Routes	A/C (%)	MDD (Kg/m^3^)	OMC (%)∖
Ado-Ijan road (Pit A)	0	1786	17.59
	2	2160	18.00
	4	2191	21.50
	6	2103	19.60
	8	2053	15.50
	10	2005	17.20
Ado-Ijan road (Pit B)	0	1794	23.30
	2	2035	20.60
	4	2134	17.60
	6	2023	22.40
	8	1945	17.40
	10	1920	23.50
Ado-Ilawe road (Pit A)	0	1734	18.20
	2	2164	15.50
	4	2040	22.00
	6	2002	19.90
	8	1993	17.90
	10	1843	19.60
Ado-Ilawe road (Pit B)	0	171	21.30
	2	2142	18.00
	4	1994	16.50
	6	1981	28.30
	8	1964	12.40
	10	1810	17.10

**Fig. 13:**
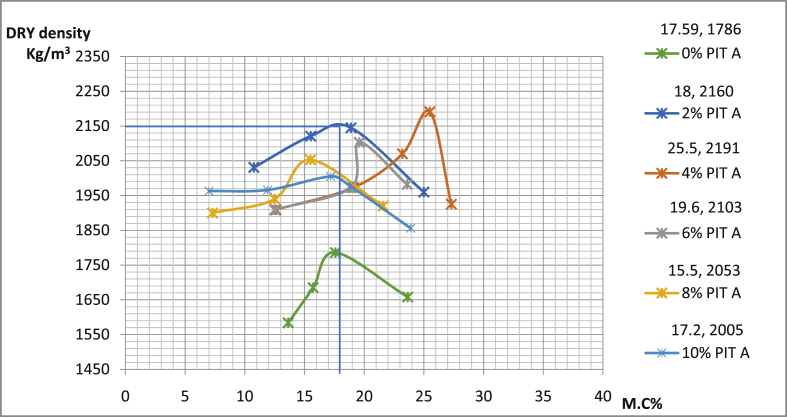
Variation of dry density with percentage moisture content for samples from Ado-Ijan road pit A.

**Fig. 14:**
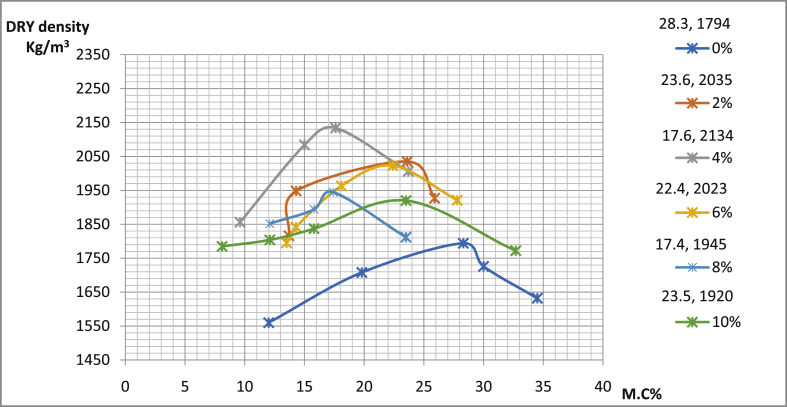
Variation of dry density with percentage moisture content for samples from Ado-Ijan road pit B.

**Fig. 15:**
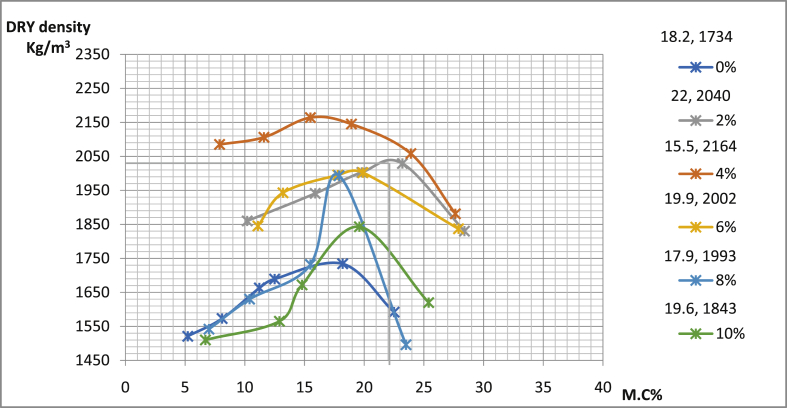
Variation of dry density with percentage moisture content for samples from Ado-Ilawe road pit A.

**Fig. 16:**
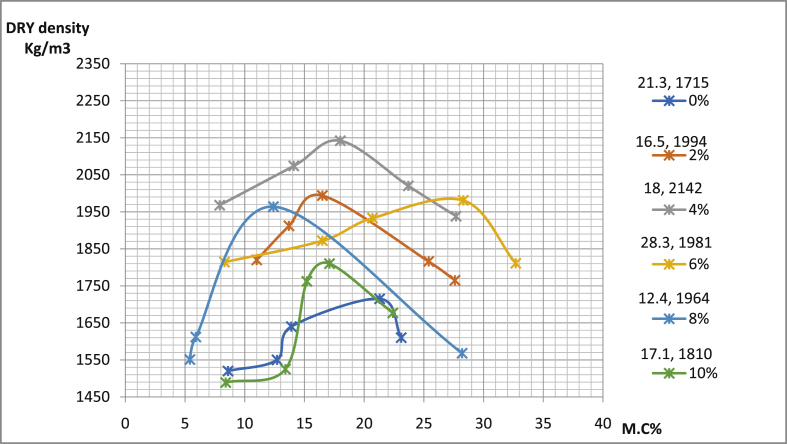
Variation of dry density with percentage moisture content for samples from Ado-Ilawe road pit B.

The plots of dry density against moisture content of the soils are shown in Fig. 13, Fig. 14, Fig. 15, Fig. 16.

### 2.5 California Bearing Ratio (CBR) test

**Fig. 17:**
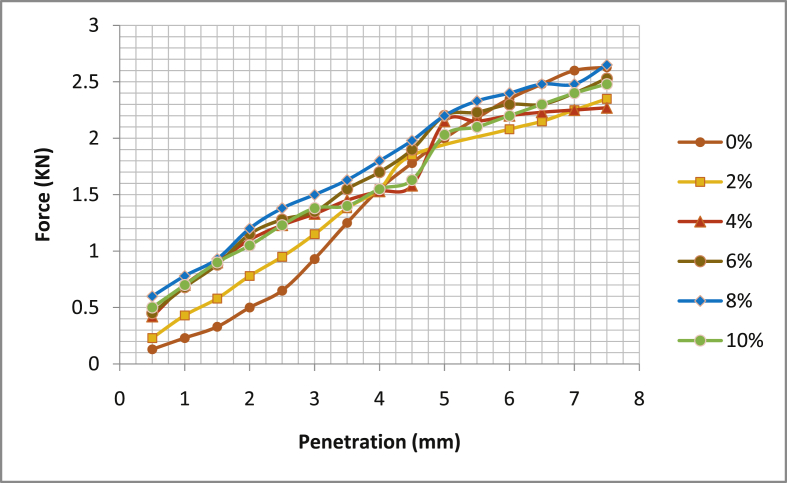
California bearing ratio graph Ado-Ijan pit A.

**Fig. 18:**
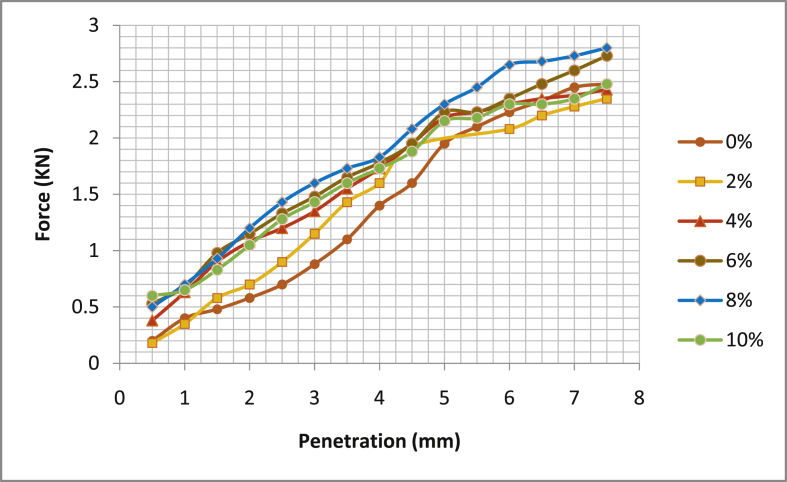
California bearing ratio graph Ado-Ijan pit B.

**Fig. 19:**
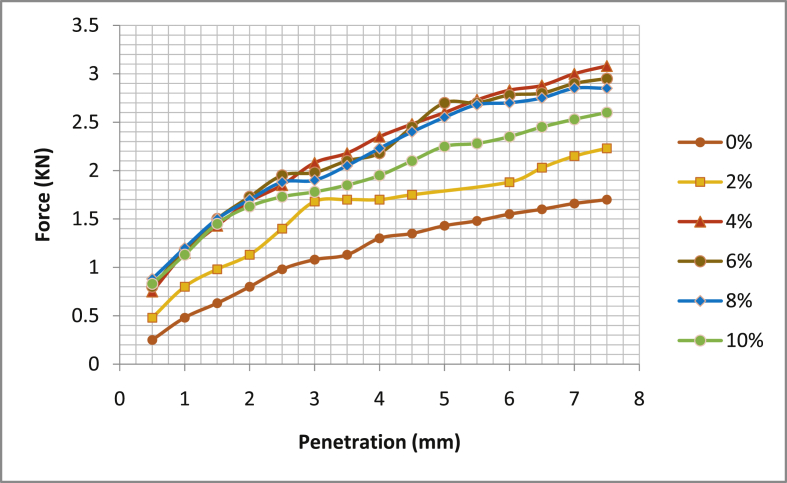
California bearing ratio graph Ado-Ilawe pit A.

**Fig. 20:**
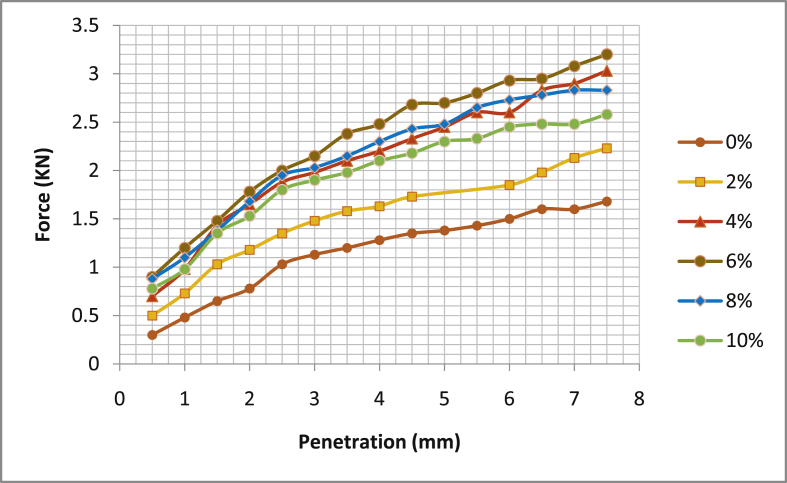
California bearing ratio graph Ado-Ilawe pit B.

Table 5 shows the summary of California Bearing Ratio test (CBR), Fig. 17, Fig. 18, Fig. 19, Fig. 20 the graphical illustration of California bearing ratio test is presented. From the table, it can be deduced that in sample location one; Ado- Ijan Pit A has its value of CBR (%) at 2.5mm and 5.0mm for 0% to be 4.91 and 10.05 respectively, but when mixed with additive it increases from 7.17 to 10.45 to 10.42 and 11.06, the same trend is observed in Pit B. In sample location two, Ado-Ilawe which has its CBR (%) at 2.5mm and 5.0mm increases from 10.57 to 9.45 to 14.73 and 13.57 when mixed with additive (CSP) while the ordinary soil is 7.4 and 7.19. It is also noted that with an increase in the percentage of the additive, the Optimum Moisture Content (OMC) increases.

**Table 5:** Summary of California bearing ratio (CBR) test.

Location		Additive content (%)	Coconut shell CBR (%) @ 2.5mm	Powder (CSP) CBR (%) @ 5.0mm
Ado-Ijan	Pit A	0	4.91	10.05
		2	7.17	10.45
		4	9.29	10.80
		6	9.67	11.06
		8	10.42	10.20
		10	9.29	10.20
	Pit B	0	5.29	9.79
		2	6.79	10.45
		4	9.06	10.95
		6	10.05	11.21
		8	10.80	11.56
		10	9.67	10.80
Ado-Ilawe	Pit A	0	7.40	7.19
		2	10.57	9.45
		4	13.97	13.07
		6	14.73	13.57
		8	14.20	12.81
		10	13.07	11.31
	Pit B	0	7.78	6.93
		2	10.19	9.29
		4	14.19	12.31
		6	15.11	13.57
		8	14.72	12.46
		10	13.60	11.56

### 2.6 Unconfined compressive strength test

From the summary of unconfined compressive strength (UCS) obtained from the laboratory as shown in Table 6, it was observed that there is increment in the strength of the soil sample with additional CSP at 2%, 4% and 6%, and reduction at 8% and 10% for Ado-Ijan pit A and pit B. For Ado-Ilawe, pit A and pit B shows increment at 2% and 4% then decreases at 6%, 8% and 10% of the additive.

**Table 6:** Summary of unconfined compressive strength test.

Percentage of additives (%)	Ado-Ijan UCS KN/m^2^		Ado-Ilawe UCS KN/m^2^	
	Pit A	Pit B	Pit A	Pit B
0	28.46	29.07	32.04	31.85
2	38.58	38.52	55.12	38.58
4	49.61	44.10	55.28	71.65
6	66.14	82.68	33.61	40.08
8	52.84	61.25	28.19	32.64
10	45.26	50.94	23.62	26.43

### 2.7 Permeability test

The summary of permeability test for Ado-ijan and Ado-ilawe location is shown in Table 7. However, the values of ‘k’ for Ado-ijan location reduces from 2.678 × 10^−3^ to 0.116 × 10^−3^for pit A. With the addition of coconut shell powder ‘CSP’ the same trend is observed in pit B while it is 2.714 × 10^−3^ and 2.737 × 10^−3^ for ordinary soil in both pits A&B, and for Ado-Ilawe the value of ‘k’ reduces from 2.66 × 10^−5^ to 0.48 × 10^−5^ for pit A and the same trend is observed in pit B with 4.27 × 10^−5^ and 4.77 × 10^−5^ with ordinary soil in pits A&B. This implies that the soil is semi pervious to pervious with variation in addition of additive (CSP) in accordance to United State Bureau of Reclamation U.S.B.R.

**Table 7:** Summary of cohesion and angle of internal friction of the soil samples.

Location		Additive content (%)	Cohesion (C) KN/m^3^	Angle of internal friction (Ø^0^)
Ado-Ijan	Pit A	0	55.33	19
		2	180.00	7
		4	132.30	9
		6	137.58	16
		8	66.00	18
		10	51.69	17
	Pit B	0	60.29	17
		2	126.91	9
		4	155.00	13
		6	54.16	23
		8	42.82	24
		10	87.56	9
Ado-Ilawe	Pit A	0	108.29	7
		2	150.61	9
		4	167.76	5
		6	85.40	13
		8	93.57	11
		10	73.79	14
	Pit B	0	104.33	9
		2	119.85	16
		4	124.68	9
		6	120.78	6
		8	104.23	9
		10	63.91	20
