# Supplementary material for: Data showing the effects of geotechnical properties of lateritic soil mixed with coconut shell powder in Ado-Ekiti, south western Nigeria
Source: Data Brief. 2019 Apr 5;24:103861. doi: 10.1016/j.dib.2019.103861 (PMC6520572; doi:10.1016/j.dib.2019.103861)

**Conflict of Interest**

*The authors have declared no conflict of interest*

**Corresponding author**

Ifetayo John OLUWAFEMI

Postgraduate School of Engineering Management, University of Johannesburg, Auckland Park Kingsway Campus, Johannesburg, 2006, South Africa

*Corresponding author: Email: [johnifetayo@gmail.com](mailto:johnifetayo@gmail.com),

Mobile: +27603797677

I/We wish to confirm that there are no known conflicts of interest associated with this publication and there has been no significant financial support for this work that could have influenced its outcome.

I/We confirm that the manuscript has been read and approved by all named author(s) and that there are no other persons who satisfied the criteria for authorship but are not listed. I/We further confirm that the order of authors listed in the manuscript has been approved by all of us.

I/We confirm that we have given due consideration to the protection of intellectual property associated with this work and that there are no impediments to publication, including the timing of publication, with respect to intellectual property. In so doing I/we confirm that I/we have followed the regulations of our institutions concerning intellectual property.

I/We understand that the Corresponding Author is the sole contact for the Editorial process (including Editorial Manager and direct communications with the office). He/she is responsible for communicating with the other authors about progress, submissions of revisions and final approval of proofs. I/We confirm that we have provided a current, correct email address which is accessible by the Corresponding Author and which has been configured to accept email from (johnifetayo@gmail.com) Ifetayo John Oluwafemi

Ifetayo John Oluwafemi


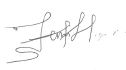

Supplement: Multimedia component 1 [file mmc1.docx]
